# Differences and commonalities of home-based care arrangements for persons living with dementia in Germany – a theory-driven development of types using multiple correspondence analysis and hierarchical cluster analysis

**DOI:** 10.1186/s12877-022-03310-1

**Published:** 2022-09-01

**Authors:** Jan Dreyer, Johannes Michael Bergmann, Kerstin Köhler, Iris Hochgraeber, Christiane Pinkert, Martina Roes, Jochen René Thyrian, Henrik Wiegelmann, Bernhard Holle

**Affiliations:** 1 Deutsches Zentrum für Neurodegenerative Erkrankungen (DZNE), site Witten, Witten, Germany; 2 Deutsches Zentrum für Neurodegenerative Erkrankungen (DZNE), site Rostock/Greifswald, Greifswald, Germany; 3 Institute for Public Health and Nursing Research, Health Sciences Bremen, University of Bremen, Bremen, Germany

**Keywords:** Family caregiver, Dementia, Alzheimer’s disease, Care arrangement, Ageing in place, Secondary analyses, Multiple correspondence analyses, Hierarchical cluster analysis, Typology

## Abstract

**Background:**

Most persons with dementia live at home and want to stay there as long as possible. In most cases, informal carers such as spouses or children care for them. Together with other family members and professional carers, they form care arrangements to address the complex needs of persons with dementia. One major aim of informal carers is to keep the care arrangement stable. The middle-range theory of ‘stability of home-based care arrangements for people living with dementia’ (SoCA-Dem theory) offers a theory to understand what constitutes and influences the stability of home-based care arrangements. Based on this theory, the aim of this study was to (1) uncover the underlying structures of differences and commonalities of home-based care arrangements for persons living with dementia, (2) construct types of these care arrangements, and (3) compare these types with regard to their stability.

**Method:**

This is a secondary analysis of data from a convenience sample of *n* = 320 care arrangements for persons with dementia obtained in the observational DemNet-D study. Data were analysed using multiple correspondence analysis and hierarchical cluster analysis. Sociodemographic data and variables related to the structure of the care arrangement (D-IVA), burden of the informal carer (BICS-D), dementia severity (FAST), and quality of life of the person with dementia (QOL-AD) were included.

**Results:**

The multiple correspondence analysis identified 27 axes that explained the entire variance between all care arrangements. The two axes ‘dementia and care trajectory’ and ‘structure of the dyadic relationship’ best distinguished care arrangements from each other and together explained 27.10% of the variance. The subsequent cluster analysis identified four types of care arrangements. Two types included spouse-centred care arrangements, and two types included child-centred care arrangements at different phases of the dementia and care trajectory. The types differ with regard to their stability.

**Conclusion:**

The results highlight the heterogeneity and commonality of care arrangements for persons living with dementia. They contribute to a better understanding of informal dementia home care. Furthermore, the results can guide the development of tailored support for persons living with dementia and their caring families.

**Supplementary Information:**

The online version contains supplementary material available at 10.1186/s12877-022-03310-1.

## Background

Most persons with dementia live at home [1] and desire to stay there as long as possible [2]. During the progression of dementia, they increasingly rely on care from other persons, which in most cases is provided by one or more informal carers. Informal carer(s) can be any people from the social network of the person with dementia who care for the person with dementia on a nonformal basis and are usually not paid. The spouse/partner or an adult child is the most common informal carer. However, other members of the social network may also care, such as neighbours, friends, grandchildren or other relatives [1]. Care by informal carers is not restricted to instrumental care but consists of several dimensions, including preventive care, supervisory care, and reciprocal care [3]. In modern societies, different forms of professional care are usually incorporated to either support the person with dementia and/or relieve the informal carer(s). This constellation of informal and formal carers involved in the care of the person with dementia and the different forms of support can be understood as a care arrangement [4].

Although these care arrangements differ noticeably from each other and change during the progression of dementia, their heterogeneity is seldom acknowledged in research [5]. In informal dementia care research, care arrangements are sometimes differentiated, for instance, by the sex or ethnicity of the informal carer or person with dementia, the kinship relation between them, or the dementia severity [6]. These (sociodemographic) categories are helpful to reveal social inequality or to obtain initial hints about differences with regard to outcomes of interventions. However, their relevance in a specific social situation is contingent; thus, their explanatory power to understand a complex social situation such as home-based care seems to be limited [7]. Furthermore, often only one (sociodemographic) category is considered, and the heterogeneity of the members representing the same category is usually high but not addressed. Nevertheless, it seems worthwhile to look for meaningful differences and commonalities between care arrangements as the acknowledgement of their heterogeneity could contribute to the development and implementation of suitable and adequate care and support structures [8] since it is commonly accepted that there is not one intervention that will work in all care arrangements [5, 8].

If meaningful differences and commonalities of home-based care arrangements are identified, they can be used to construct different types of care arrangements. These types can be used to bridge the complexity of a single case and the tendency towards uninformative generalisations based on average values of the whole population [9]. Depending on the research question and underlying theory, a potentially very large number of variables could be considered to distinguish between care arrangements. This study is part of the ‘stability of care arrangements for persons living with dementia’ (SoCA) research project of the Deutsches Zentrum für Neurodegenerative Erkrankungen (DZNE), site Witten. Within this project, we investigate the stability of home-based care arrangements for persons living with dementia. Informal carers strive to stabilise their care arrangement [10]. A care arrangement is stable if the person with dementia can stay at home and his or her needs and the needs of the informal carer are addressed adequately [11]. Care arrangements differ with regard to their stability [12]. In some care arrangements, the person with dementia moves relatively quickly into a long-term care facility or stays at home under unsustainable conditions for herself/himself or the informal carer. In contrast, in other care arrangements and under conditions that are adequate for the person with dementia and the informal carer, the person with dementia is able to stay at home for several years. Many care arrangements oscillate between these two poles over the often long-lasting care trajectory [13, 14].

**Fig. 1:**
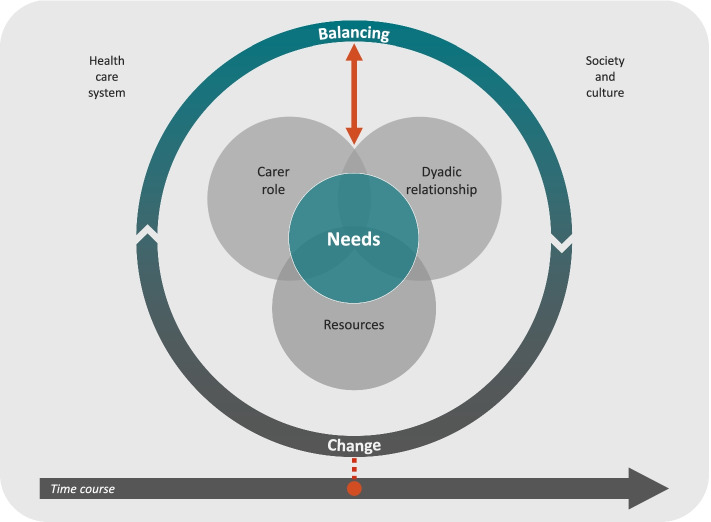
Conceptual model of stability of home-based care arrangements for persons living with dementia. (SoCA-Dem theory) [15] (The original model was published under CC BY-NC (https://creativecommons.org/licenses/by-nc/4.0/), we adapted the colour scheme)

To understand how stability is constituted and influenced, we developed a middle-range theory of the stability of home-based care arrangements for persons living with dementia (SoCA-Dem theory) [15]. Within this theory (Fig. 1), we define the cyclic process of the concepts of *change* and *balancing* as pivotal to understanding the stability of care arrangements. Over the course of time, the informal carer(s) must continuously react to changes caused by the progression of the dementia or changes that occur from other sources and balance their consequences. These actions of the carer(s) are influenced by the concepts of the *dyadic relationship* (e.g., the kind of the relationship between the person with dementia and the carer), *carer role* (e.g., the motivation of the informal carer to provide care), *resources* (e.g., income or the amount of social support) and *needs* (e.g., the need for social inclusion) in the centre of the model. The entire process is embedded in the social context formed by the concepts of the *health care system* (e.g., the availability and affordability of health care services) and *society and culture* (e.g., social values and expectations). Depending on the formation of each concept of the SoCA-Dem theory, care arrangements differ in their stability.

The SoCA-Dem theory is the underlying theory of the present study and guides the selection of variables to investigate differences and commonalities and to construct types of care arrangements. This study is the first attempt to translate the theoretical concepts of the SoCA-Dem theory into measurable variables, which is the prerequisite to make the SoCA-Dem theory empirically accessible for quantitative research.

The present study of the SoCA-Dem research project aims to answer the following research questions:

1. Based on the SoCA-Dem theory, what are the underlying structures of differences and commonalities of home-based care arrangements for persons living with dementia?
2. Which types of care arrangements could be constructed by means of these differences and commonalities?
3. Do these types of care arrangements differ in their stability?

## Methods

### Study design

This is a secondary, explorative analysis of data gathered in the observational, multicentre, longitudinal (one-year follow-up) DemNet-D study (2012–2015). The DemNet-D study aimed to evaluate dementia care networks (DCNs) in Germany [16].

### Sample and data collection

The DemNet-D study was based on a convenience sample of 560 persons with dementia and their carers from 13 DCNs across Germany. Trained interviewers collected data in face-to-face interviews with the informal carers. Details about the sampling procedure and data collection are reported elsewhere [17, 18].

For this secondary analysis, only care arrangements in which the person with dementia lived in a private household and was cared for by an informal carer were included. Furthermore, only care arrangements without missing values were included (see Table 1 for inclusion and exclusion criteria).

**Table 1:** Inclusion and exclusion criteria

Inclusion criteria	Exclusion criteria
One of the following answer categories of the variable ‘living situation of the person with dementia’: • Cohabiting with spouse/partner in same household • Living alone in own household • Cohabiting with adult children (in-law) in same household • In a relative’s place but in own independent household • Cohabiting with other relative in same household • Living alone in own household with live-in carer	One of the following answer categories of the variable ‘living situation of the person with dementia’: • Shared flat for persons with dementia • Assisted living facility • Nursing home • Missing value
One of the following answer categories of the variable ‘kinship relation between the person with dementia and the carer’: • Spouse • Child • Child-in-law • Grandchild • Other family member • Friend • Neighbour	One of the following answer categories of the variable ‘kinship relation between the person with dementia and the carer’: • Professional carer • Legal guardian • Missing value
Complete cases regarding the active variables	Missing values regarding the active variables

### Measurements

In the DemNet-D study, an extensive questionnaire consisting of several standardised instruments and specifically developed questions was used to assess care arrangements for persons living with dementia at home [16]. To operationalise the SoCA-Dem theory, the DemNet-D-variables were chosen (by JD) that best represented the concepts of the SoCA-Dem theory and were defined as active variables. Additionally, variables that were fit to serve as indicators for the stability of home-based care arrangements were defined (by JD) as passive variables. These preliminary selections were discussed within the SoCA research team (JD, KK, IH, CP, BH) until a final set of 25 variables was included. Since multiple correspondence analysis (MCA) required categorical variables, continuous variables (e.g., age) were converted into categorical variables. Furthermore, the number of possible answer categories should be limited, and categories with extremely low response rates should be avoided [19]. Therefore, categories were computed in such a way that variables had no more than three answer categories. The included active variables and their assignment to the concepts of the SoCA-Dem theory are displayed in Table 2. The underlying variables of the primary DemNet-D study are presented in Additional file 1: Appendix A. The selected variables originate from sociodemographic questions, the Instrument for Assessing Home-Based Care Arrangements for Persons with Dementia (D-IVA) [12], the Berlin Inventory of Caregiver Stress—Dementia (BICS-D) [20], the Functional Assessment Staging (FAST) tool [21] and the Quality of Life in Alzheimer's Disease (QOL-AD proxy) questionnaire [22].

#### Active variables

**Fig. 2:**
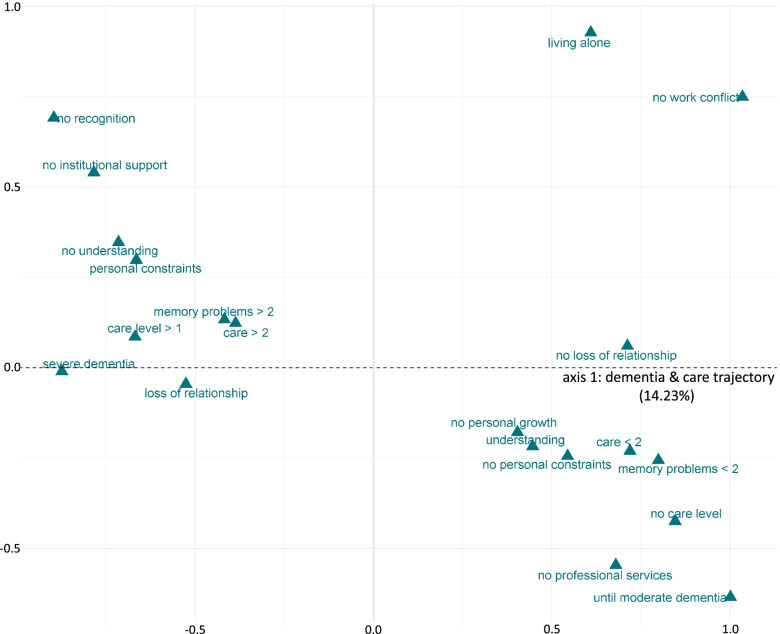
MCA map of the contributing categories to the axis ‘dementia and care trajectory’

**Fig. 3:**
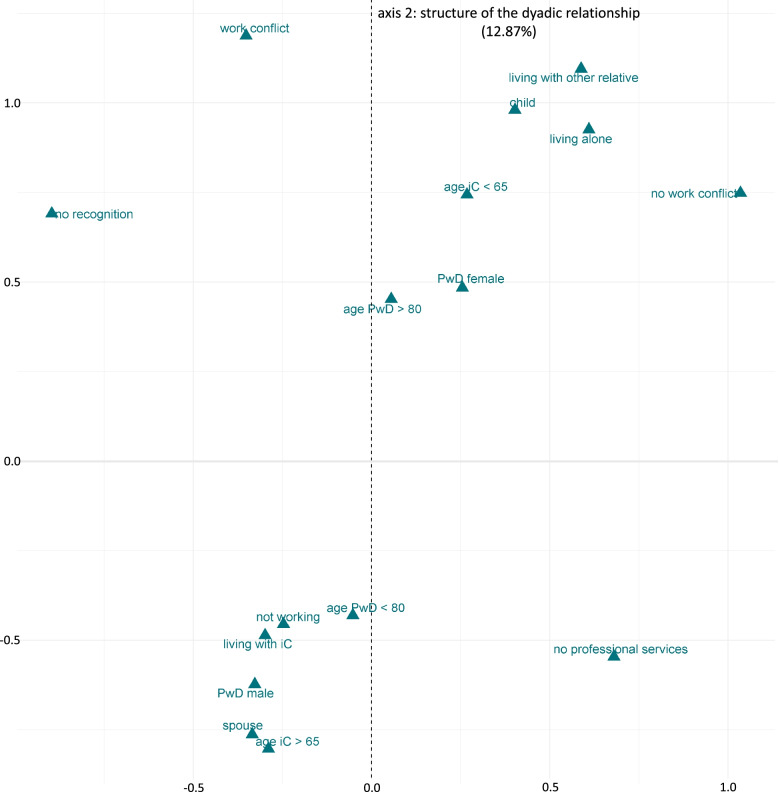
MCA map of the contributing categories to the axis ‘structure of the dyadic relationship’

**Fig. 4:**
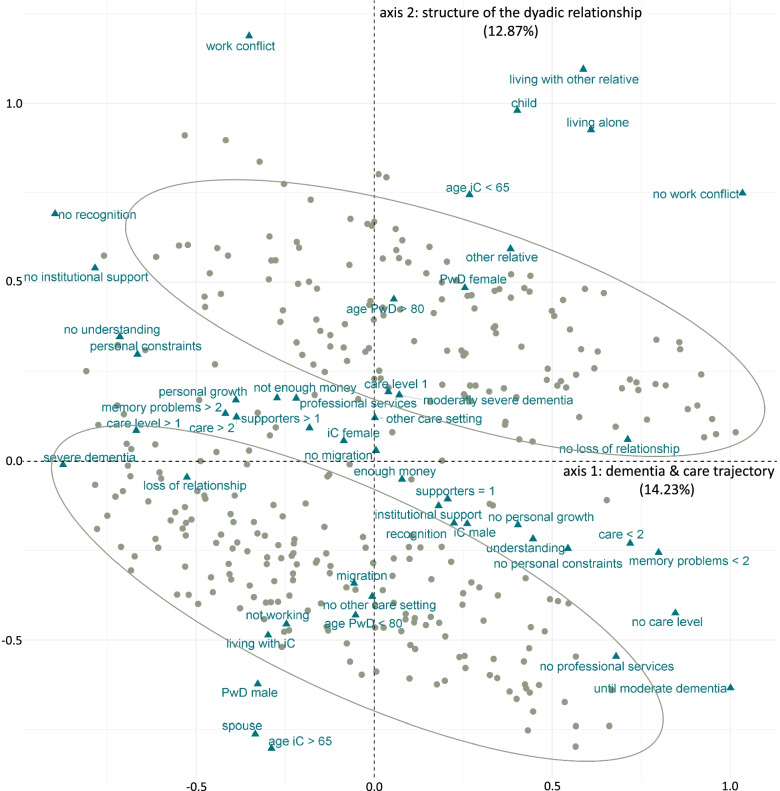
MCA map for the superimposed representation of care arrangements and categories

Overall, 22 active variables with 49 answer categories were included. For the concepts of *balancing* and *needs* of the SoCA-Dem theory, no suitable variables could be identified in the DemNet-D dataset. For all other concepts, between 1 and 5 variables were selected. All active variables, their categories and short names are displayed in Table 2. The short names are used in Figs. 2, 3 and 4 to display the distribution of the categories in the geometrical space of the MCA.

**Table 2:** Active variables and their categories

Concept SoCA	Variable	Categories	Short names
**Dyadic Relationship**	kinship relation between iC and PwD	spouse/partner	spouse
		parent–child	child
		other relative	other relative
	living situation of the PwD	living alone (or alone with 24 h help)	living alone
		cohabiting with iC	living with iC
		cohabiting with another relative	living with other relative
	loss of relationship between the PwD and the iC	often/always	loss of relationship
		never/sometimes	no loss of relationship
	age of the iC	up to 65 years old	age iC < 65
		older than 65 years old	age iC > 65
	age of the PwD	up to 80 years old	age PwD < 80
		older than 80 years old	age PwD > 80
**Change**	care level of the PwD according to German long-term care insurance^a^	no care level	no care level
		first care level	care level 1
		second or third care level	care level > 1
	functional/cognitive ability of the PwD (FAST)	until moderate Alzheimer’s	until moderate dementia
		moderately severe Alzheimer’s	moderately severe dementia
		severe Alzheimer’s	severe dementia
	period since the PwD needed help because of the memory loss	since up to 2 years	memory problems < 2
		since more than 2 yeas	memory problems > 2
	period since the iC cared for the PwD	since up to 2 years	caring < 2
		since more than 2 years	caring > 2
**Carer Role**	role conflict between caring for the PwD and profession	always/often	work conflict
		never/sometimes	no work conflict
		not working	not working
	the iC experiences personal constraints due to caring	always/often	personal constraints
		never/sometimes	no personal constraints
	the iC experiences personal growth due to caring	always/often	personal growth
		never/sometimes	no personal growth
	the iC experiences no recognition from others	always/often	recognition
		never/sometimes	no recognition
	the iC could imagine PwD moving to institutional care	yes	other care setting
		no	no other care setting
**Resources**	number of professional services used	none	no professional services
		as least one	professional services
	number of groups of informal supporters	one	supporters = 1
		two or more	supporters > 1
	the PwD or the iC has sufficient financial resources	yes	enough money
		no	not enough money
**Society & Culture**	gender of the iC	female	iC female
		male	iC male
	gender of the PwD	female	PwD female
		male	PwD male
	migration background of the iC	yes	migration
		no	no migration
	the iC experiences too little understanding from others for PwD	always/often	no understanding
		never/sometimes	understanding
**Health Care System**	the iC experiences a lack of institutional support	always/often	no institutional support
		never/sometimes	institutional support

*iC* Informal carer, *PwD* Person with dementia

^a^ the care level describes the need for care of the PwD according to the German long-term care insurance. Here, the older model with care levels ranging from 1 (lowest) to 3 (highest) was used

#### Passive variables

Three passive variables were included in the analyses as indicators for the stability of the home-based care arrangements. All passive variables and their categories are displayed in Table 3.

**Table 3:** Passive variables and their categories

Variable	Categories
care situation from the perspective of the informal carer at t0	caring at home does not work anymore
	the care situation is well organised, but in case of the progression of dementia, more help is needed
	the care situation is well organised; even if the dementia progresses, more help is not needed
	missing
quality of life of the person with dementia at t0	range from 13 (worst quality of life) to 52 (best quality of life)
living situation of the person with dementia at t1	still private home
	moved to an institutional form of living
	died
	missing

### Statistical analyses

We used multiple correspondence analysis (MCA) and hierarchical cluster analysis (HCA) to reveal underlying structures of the care arrangements and to construct types of care arrangements for persons living with dementia. Both analytical methods were explorative and data structuring and therefore required decisions during the process of analysis. These decisions were made after discussion within the research team (JD, KK, IH, CP, BH, MR) and an expert in MCA and HCA (JoB). A detailed description of the chosen analytical approach in the context of health care has been published elsewhere [23, 24].

#### Multiple correspondence analysis

As a first step, we performed MCA. The aim of MCA is to analyse the relationships between the categories of the included variables [25]. For this purpose, MCA calculates the singular value decomposition of the complete disjunctive table, yielding a set of eigenvalues (called variance in the results section) and corresponding eigenvectors (called axes in the results section). The eigenvalues represent synthetic quantitative variables that summarise all categorical variables [25]. To interpret the eigenvalues, it is possible to calculate the contribution of every category of the variables to every eigenvalue [26]. The relationships between the categories and between the care arrangements can be visualised in a geometrical space to guide the interpretation of the results of MCA. For this purpose, the chi-square distances are interpreted as Euclidean distances and plotted in the geometrical space formed by the eigenvectors. Small distances indicate similarity between categories or between care arrangements; large distances indicate dissimilarity. MCA is a dimension-reducing procedure that selects a few characteristic combinations from the many possible characteristics in such a way that as much information as possible is retained from the data. This reduction in complexity is a guiding principle of the MCA that helps to interpret the underlying structure of the data. Therefore, usually only the first two or three dimensions are visualised as a geometrical space. Consequently, the Euclidean distances between categories or between care arrangements seem to be smaller than they are in high-dimensional datasets such as ours [27]. All active variables and their categories are used to calculate the eigenvalues and their corresponding eigenvectors. The passive variables are not used to perform these calculations and therefore have no influence on the distances between categories or between care arrangements but can be plotted in the same geometrical space [25].

#### Hierarchical cluster analysis

In a subsequent step, HCA was performed to cluster the care arrangements based on the Euclidean distances between the home-based care arrangements computed in MCA. This means that the care arrangements were clustered by using their Euclidean distances to one another in the geometrical space of MCA. As recommended by Husson, Lê and Pagès [26], we included not all eigenvalues of MCA for HCA but the eigenvalues that represent a summarised variance of 80% to 90% of the overall variance only. The remaining eigenvalues were interpreted as noise that should not be considered to construct stable clusters. To cluster the care arrangements, we used the Ward method to minimise the variance within the clusters and maximise the variance between the clusters [28]. The number of clusters was determined first on the basis of interpretability and second on statistical reasons (elbow criteria). As a measurement of the validity of the final cluster solution, we used the silhouette coefficient [29]. Following HCA, we applied the v-test to check the extent to which the categories corresponded to the identified clusters. The v-test compared the proportion of the category in a cluster to the proportion of the category in the whole dataset. With this test, we identified significant categories (p < 0.05) that described the clusters [26].

#### Handling of missing data

Multiple correspondence analysis requires complete cases. We judged the missing data as not missing at random and therefore avoided the imputation of missing data and decided to include complete cases only [30]. As passive variables have no influence on the MCA, missing data in these variables were accepted.

#### Software

The selection and editing of variables were performed with IBM SPSS Statistics version 21. MCA and HCA were performed with R version 3.6.3 [31] and the R package FactoMineR [32].

#### Reporting guideline

The STROBE *checklist* was used to write this report [33].

## Results

### Participants

A total of 320 care arrangements of the 560 care arrangements of the DemNet-D study fulfilled the inclusion criteria of the present study and were included in the analyses. A total of 240 care arrangements were excluded due to the living situation of the PwD (*n* = 44), the kinship relation between the PwD and the iC (*n* = 19), or both (*n* = 12) and a missing value in at least one active variable (*n* = 165). The demographic data of the person with dementia and the informal carer of the included 320 care arrangements are displayed in Table 4.

**Table 4:** Sample characteristics

Sample size		*n* = 320 (100)^a^
Sex of the informal carer	female	241 (75.3)
Age of the informal carer		64.53^b^ [SD 12.5], [24–93]^c^
Sex of the person with dementia	female	180 (56.3)
Age of the person with dementia		79.62^b^ [SD 8.17], [44–101]^c^
Kinship relation between the person with dementia and the informal carer	spouse child child-in-law friend grandchild other family member	174 (54.4) 119 (37.2) 16 (5) 3 (0.9) 3 (0.9) 5 (1.6)
Living situation of the person with dementia	cohabiting with spouse/partner living alone in own household cohabiting with adult children (in-law) in a relative’s place but in own household cohabiting with other relative living alone in own household with live-in carer	182 (56.9) 70 (21.9) 37 (11.6) 25 (7.8) 5 (1.6) 1 (0.3)
Diagnosis of dementia by a physician	yes no missing	292 (91.3) 21 (6.6) 7 (2.2)
Dementia type	Alzheimer’s dementia vascular dementia fronto-temporal dementia dementia with Lewy bodies Parkinson’s dementia unspecific missing	124 (38.8) 59 (18.4) 3 (0.9) 1 (0.3) 4 (1.3) 89 (27.8) 40 (12.5)
Functional Assessment Staging (FAST)	normal adult normal aged adult incipient Alzheimer’s Disease mild Alzheimer’s Disease moderate Alzheimer’s Disease moderately severe Alzheimer’s Disease severe Alzheimer’s Disease	1 (0.3) 5 (1.6) 1 (0.3) 21 (6.6) 26 (8.1) 189 (59.1) 77 (24.1)
Care level according to the German long-term care insurance^d^	none care level 1 care level 2 care level 3 applied for, not decided applied for, not approved	54 (16.9) 129 (40.3) 92 (28.7) 18 (5.6) 20 (6.3) 7 (2.2)

*SD* Standard deviation

^a^ numbers in brackets = relative frequencies in percent; ^b^ arithmetic mean; ^c^ = range; ^d^ the care level describes the need for care of the PwD according to the German long-term care insurance. Here, the older model with care levels ranging from 1 (lowest) to 3 (highest) was used

### Relations between the characteristics of the care arrangements: results of multiple correspondence analysis

The overall variance of the data was 1.227 and could be displayed on 27 axes. On average, every axis explained 3.70% of the overall variance. The two axes with the highest explained variance explained 27.10% of the overall variance. The third axis explained 6.82% of the overall variance only, and the following axes decreased regularly with only small differences (see Additional file 1: appendix B). Therefore, we decided to interpret only the two axes with the highest explained variance in detail. To interpret them, we considered all categories that contributed above the average contribution per category of 2.04%.

The first axis explained 14.23% of the overall variance. The categories of the concept *change* of the SoCA-Dem theory contributed the most to this axis at 33.9%, followed by the categories of the concept *carer role* (21.4%) and *dyadic relationship* (11.9%) (see Table 5).

**Table 5:** Contribution of categories to the first and second axes

	**Axis 1: dementia & care trajectory**			**Axis 2: structure of the dyadic relationship**		
	**Category**	**Contribution** **to axis**		**Category**	**Contribution to axis**	
**change**	memory problems < 2 years severe dementia care < 2 years no care level until moderate dementia care level > 1 memory problems > 2 care > 2	5.71% 4.78% 4.71% 4.71% 4.40% 4.01% 2.99% 2.54%	**33.9%**^**a**^			
**dyadic relationship**	no loss of relationship loss of relationship living alone	5.61% 4.15% 2.14%	**11.9%**	child spouse age iC > 65 age iC < 65 living alone living with the iC living with other relative age PwD > 80 age PwD < 80	10.29% 9.10% 8.92% 8.28% 5.48% 4.56% 3.78% 2.88% 2.74%	**56.0%**
**carer role**	no work conflict personal constraints no personal constraints no recognition no personal growth	5.66% 5.19% 4.24% 4.19% 2.08%	**21.4%**	work conflict not working no work conflict no recognition	5.20% 3.99% 3.28% 2.75%	**15.2%**
**other categories above average**	no understanding understanding no institutional support no professional services	5.11% 3.19% 3.00% 2.93%	**14.2%**	PwD male PwD female no professional services	4.89% 3.80% 2.09%	**10.8%**
**categories below average**	29 categories		**18.6%**	33 categories		**18.0%**

*Ic* Informal carer, *PwD* Person with dementia; the short names of the categories are explained in Table 2

^a^Bold numbers indicate the cumulative contribution of all categories of the respective concept of the SoCA-Dem theory

In the following, we interpret the first two axes one by one. In Fig. 2, axis 1 and all categories that contributed above the average to this axis are displayed in a coordinate system (please see Table 2 for the short names of the categories). On the left side are, for example, the categories *the iC cares for the PwD longer than two years*, *the PwD has severe dementia*, and *the iC always or often experiences personal constraints*. On the right side, there are opposing categories, such as *the iC cares for the PwD for less than two years*, *the PwD has moderate dementia* and *the iC never or sometimes experiences personal constraints*. In sum, axis 1 opposes categories that describe care arrangements at later stages of the care trajectory with more care dependency of the person with dementia and more role conflicts of the informal carer (left side of Fig. 2) against categories that describe care arrangements at the beginning of the care trajectory with less care dependency of the person with dementia and fewer role conflicts of the informal carer (right side of Fig. 2). Therefore, we named this axis the ‘dementia and care trajectory’.

The second axis explained 12.87% of the overall variance. The categories of the concept *dyadic relationship* of the SoCA-Dem theory contributed the most to this axis at 56.0%, followed by the categories of the concept *carer role* (15.2%) (see Table 5). In Fig. 3, the second axis and all categories that contributed above the average to the second axis are displayed (please see Table 2 for the short names of the categories). At the top are, for example, the categories *the iC is the child of the PwD*, *the iC is 65 years or younger*, and *the PwD is living alone*. At the bottom are opposing categories, such as *the iC is the spouse or partner of the PwD*, *the iC is older than 65 years* and *the PwD is cohabiting with the iC*. In sum, axis 2 opposes categories that describe care arrangements in which a non-cohabiting child of the person with dementia is the informal carer (at the top of Fig. 3) against categories that describe care arrangements in which a cohabiting spouse is the informal carer (at the bottom of Fig. 3). Therefore, we named this axis ‘structure of the dyadic relationship’.

Both axes are used to construct a two-dimensional space that explains 27.10% of the variance (Fig. 4). All categories (triangles) and all included individual care arrangements (points) of the sample are projected in this space. Distances between categories or between care arrangements can be interpreted as dissimilarity if the distance is large or as similarity if the distance is short. However, these distances must be interpreted with caution as they appear to be smaller because the distances on the other 25 axes are not displayed in this two-dimensional figure. The quality of representation for all categories for all axes is displayed in Additional file 1: Appendix C. It is notable that the distribution of care arrangements corresponds with the distribution of the categories. There are two groups of care arrangements (framed by the ellipses) that are separated by the axis *structure of the dyadic relationship*. Both groups are oriented along the axis *dementia and care trajectory*.

### Types of care arrangements: results of the hierarchical cluster analysis

HCA is based on the distances between the care arrangements of the first 15 axes, which explain 81.23% of the overall variance. The elbow criterion suggested differentiation into four types (see Additional file 1: Appendix D). This solution also had the best interpretability and was therefore chosen. The four types explain 24.08% of the variance. The silhouette value for this solution is 0.13, and the silhouette plot is displayed in Additional file 1: Appendix E. Due to the explorative character of this study and its large context heterogeneity, we interpret these validation measures as acceptable.

**Fig. 5:**
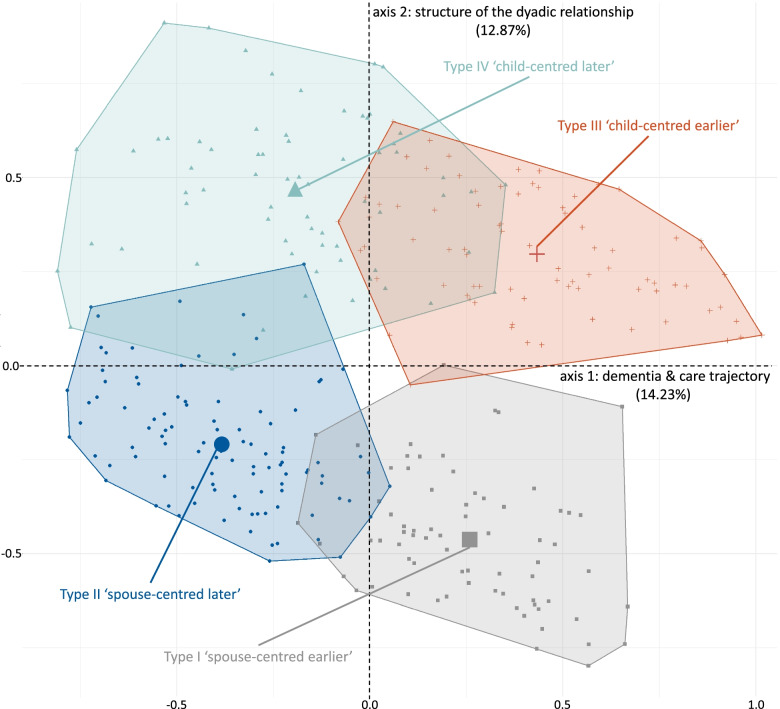
Types of care arrangements in the correspondence space of the MCA

The four types are displayed in the correspondence space of MCA in Fig. 5. The care arrangements are coloured and formed according to their assignment to one of the four types. The axis ‘dementia and care trajectory’ separates the two types on the left side from the two types on the right side. This suggests that the two types on the left side are similar in relation to *the dementia and care trajectory* and differ from the two types on the right side. The axis ‘structure of the dyadic relationship’ separates the two types at the top from the two types at the bottom. This suggests that the types at the top are similar in their *structure of the dyadic relationship* and differ from the two types at the bottom. The overlapping of the types is a result of the projection in the two-dimensional space and is only a supposed overlap.

#### Description of the four identified types

In the following, we describe the types by means of the categories used for MCA that differ significantly between the respective type and the whole sample. The distribution of all categories of the active variables for the whole sample and for the four types are displayed in Table 6.

**Table 6:** Distribution of categories in relation to the types

Concept of the SoCA-Dem theory	Variable	Categories	Whole sample	Type I: spouse-centred earlier	Type II: spouse-centred later	Type III: child-centred earlier	Type IV: child-centred later
size			100 (*n *= 320)	22.50 (*n* = 72)	31.88 (*n* = 102)	24.69 (*n* = 79)	20.94 (*n* = 67)
**Dyadic Relationship**	kinship relation between the iC and the PwD	spouse/partner parent–child other relative	54.38 37.19 8.447	94.44*** 2.78*** 2.78*	96.08*** 0.98*** 2.94*	1.27*** 81.01*** 17.72**	10.45*** 77.61*** 11.94
	living situation of the PwD	living alone cohabiting with the iC cohabiting with another relative	22.19 66.88 10.94	0.00*** 98.61*** 1.39**	1.96*** 97.06*** 0.98**	62.03*** 15.19*** 22.79**	29.85 47.76** 22.39**
	loss of relationship between the PwD and the iC	often/always never/sometimes	57.50 42.50	40.28** 59.72**	87.26*** 12.75***	30.38*** 69.62***	62.69 37.31
	age of the iC	up to 65 years old older than 65 years old	51.88 48.13	18.06*** 81.94***	13.73*** 86.28***	94.94*** 5.06***	95.52*** 4.48***
	age of the PwD	up to 80 years old older than 80 years old	51.25 48.75	73.61** 26.39**	64.71** 35.29**	29.11*** 70.89***	32.84** 67.16**
**Change**	care level of the PwD according to the German long-term care insurance	no care level first care level second or third care level	25.31 40.31 34.38	51.39*** 33.33 15.28**	6.86*** 39.22 53.92***	35.44* 46.84 17.72**	13.43* 41.79 44.78*
	functional/cognitive ability of the PwD (FAST)	up to moderate Alzheimer’s moderately severe Alzheimer’s severe Alzheimer’s	16.88 59.06 24.06	40.28*** 50.00 9.72**	4.90** 52.94 42.16***	24.05 68.35 7.60**	1.49** 67.16 31.34
	period since the PwD needs help because of the memory loss	since up to 2 years since more than 2 years	34.38 65.63	77.78*** 22.22***	2.94*** 97.06***	41.77 58.23	26.87 73.13
	period in which the iC cares for the PwD	since up to 2 years since more than 2 years	35.00 65.00	76.39*** 23.61***	4.90*** 95.10***	41.77 58.23	28.36 71.62
**Carer Role**	role conflict between caring for the PwD and profession	always/often never/sometimes not working	12.81 20.31 66.88	2.78** 5.56** 91.67***	0.98*** 0.98*** 98.04***	2.53** 69.62*** 27.85***	53.73*** 7.46** 38.81***
	the iC experiences personal constraints due to caring	always/often never/sometimes	45.00 55.00	15.28*** 84.72***	62.75** 37.26**	13.92*** 86.08***	85.57*** 13.43***
	the iC experiences personal growth due to caring	always/often never/sometimes	50.94 49.06	33.33** 66.67**	62.75** 37.26**	40.51* 59.49*	64.18* 35.82*
	the iC experiences no recognition from others	always/often never/sometimes	20.00 80.00	0.00*** 100.00***	24.51 75.49	2.53*** 97.47***	55.22*** 44.78***
	the iC could imagine the PwD moving to institutional care	yes no	75.63 24.38	65.28* 34.72*	73.53 26.47	82.28 17.72	82.09 17.91
**Resources**	number of professional services used	none as least one	24.38 75.63	52.78*** 47.22***	11.77** 88.24**	25.32 74.68	11.94** 88.06**
	number of groups of informal supporters	one two or more	46.88 53.13	62.50** 37.50**	39.22 60.78	51.90 48.10	35.82* 64.18*
	the PwD or the iC has sufficient financial resources	yes no	77.81 22.19	87.50* 12.50*	72.55 27.45	87.32* 12.66*	64.18** 35.82**
**Society & Culture**	gender of the iC	female male	75.31 24.69	66.67 33.33	73.53 26.47	73.42 26.58	89.55** 10.45**
	gender of the PwD	female male	56.25 43.75	34.72** 65.28**	30.39*** 69.61***	86.08*** 13.92***	83.58*** 16.42***
	migration background of the iC	yes no	8.13 91.88	15.28* 84.72*	5.88 94.12	5.06 94.94	7.46 92.54
	the iC experiences too little understanding of others for the PwD	always/often never/sometimes	38.44 61.56	11.11*** 88.89***	52.94** 47.06**	13.92*** 86.08***	74.63*** 25.37***
**Health Care System**	the iC experiences a lack of institutional support	always/often never/sometimes	18.75 81.25	2.78** 97.22**	23.53 76.47	7.60** 92.41**	41.79*** 58.21***

Numbers indicate relative frequencies

*iC*  Informal carer, *PwD*  Person with dementia

^*^indicate significant categories for corresponding types (*p < 0.05, **p < 0.01, ***p < 0.00001)

Type I (the grey polygon in Fig. 5) is named ‘spouse-centred care arrangements at the earlier stages of the dementia and care trajectory’ (hereafter abbreviated ‘spouse-centred earlier’). A total of 22.50% of all care arrangements were assigned to this type. In this type, almost all carers were the spouse of the person with dementia (94.44%), were older than 65 years (81.94%) and did not work (91.67%). The persons with dementia were mostly younger than 80 years (81.94%), more often male (65.28%) and lived together with the carer (98.61%). Furthermore, most persons with dementia received care and support for less than two years (77.78%), only 15.28% had a care level above one, and only 9.72% had severe dementia, indicating a rather low care dependency of the persons with dementia. Almost all carers experienced no personal constraints due to caring (84.72%). All carers never or only sometimes experienced a lack of recognition from others (100.00%). In half of the care arrangements, no professional services were incorporated (52.78%), and only one group of informal supporters (62.5%) was involved. Nevertheless, most carers (97.22%) did not experience a lack of institutional support or too little understanding from others for the person with dementia (88.89%).

Type II (the blue polygon in Fig. 5) is named ‘spouse-centred care arrangements at the later stages of the dementia and care trajectory’ (hereafter abbreviated ‘spouse-centred later’). A total of 31.80% of all care arrangements were assigned to this type. Almost all carers were the spouse of the person with dementia (96.08%), were older than 65 (86.28%) and did not work (98.04%). Almost all persons with dementia cohabitated with the informal carers (97.06%), were more often male (69.61%) and were younger than 80 (64.71%). Half of them had the second or third care level (53.90%) and severe dementia (42.16%). These categories indicate a high care dependency of the persons with dementia. Almost all persons with dementia needed help due to loss of memory for more than two years (97.06%). In most care arrangements, professional services (88.24%) and two or more groups of informal supporters (60.78%) were involved. Half of the carers perceived little understanding from others for the person with dementia (52.94%), and many experienced personal constraints due to the provision of care and support (62.75%) as well as a loss of relationship to the person with dementia (87.26%). In addition to these negative consequences, many carers experienced personal growth due to caring (62.75%).

Type III (the red polygon in Fig. 5) is named ‘child-centred care arrangements at the earlier stages of the dementia and care trajectory’ (hereafter abbreviated ‘child-centred earlier’). A total of 24.69% of all care arrangements were assigned to this type. The structure of this dyadic relationship is very different from the first two types: most carers were a child of the person with dementia (81.01%) and younger than 65 years (94.94%). The persons with dementia were mostly older than 80 years (70.89%), female (86.08%) and frequently lived alone (62.03%) or together with a relative other than the informal carer (22.79%). They had no (35.44%) or the first care level (46.84%) and seldom had severe dementia (7.60%). These categories indicate a moderate care dependency of the persons with dementia. A total of 72.15% of the carers were employed, but only 2.53% experienced a role conflict between caring and working. Furthermore, most carers never or sometimes experienced personal constraints (86.08%), a lack of institutional support (92.41%) or no recognition (97.04%). In many care arrangements, formal services were involved (74.68%).

Type IV (the mint polygon in Fig. 5) is named ‘child-centred care arrangements at the later stages of the dementia and care trajectory’ (hereafter abbreviated ‘child-centred later’). A total of 20.94% of all care arrangements were assigned to this type. Most carers were a child of the person with dementia (77.61%), younger than 65 (95.52%) and female (89.55%). The persons with dementia were also mostly female (83.58%) and older than 80 years (67.16%). Two-thirds of them cohabited with the carer (47.76%) or another relative (22.39%), but a third lived alone (29.85%). Half of them had the second or third care level (44.78%), and one-third had severe dementia (31.34%). Both categories indicate a high care dependency of the persons with dementia. In many care arrangements, professional services (88.06%) and more than one group of informal supporters (64.18%) were present. Most carers perceived personal constraints (85.57%) and too little understanding from others for the person with dementia (74.63%). Half of them experienced no recognition from others (55.22%), role conflicts between caring and working (53.73%) and a lack of institutional support (41.79%). Furthermore, a third stated that they have not sufficient financial resources (35.82%). Despite these challenging experiences, almost two-thirds of the carers experienced personal growth due to caring (64.18%).

#### Relationship between the four identified types and the indicator variables for stability

In this section, we compare the types of care arrangements in relation to the passive variables to determine whether the types differ in their stability (see Table 7).

**Table 7:** Distribution of the passive variables in relation to the types

Variable	Categories	Whole sample	Type I: spouse-centred earlier	Type II: spouse-centred later	Type III: child-centred earlier	Type IV: child-centred later
care situation from the perspective of the iC at t0	caring at home does not work anymore	10.00	0.00**	10.78	10.13	19.40**
	the care situation is well organised, but in case of progression of the dementia, more help is needed	58.44	50.00	60.78	65.82	55.22
	the care situation is well organised; even if the dementia progresses more help is not needed	28.44	45.83**	23.53	21.52	25.37
	missing	3.13	4.17	4.90	2.53	0.00
quality of life of the PwD at t0		28,52^a^ (SD 5.21) [16-48]	32.49*** (SD 4.88) [21-48]	26.77** (SD 4.57) [16-41]	29.28 (SD 4.70) [20-39]	26.02** (SD 4.26) [17-35]
living situation of the PwD at t1	private home	64.38	80.56**	62.75	53.17*	62.69
	PwD moved to an institutional form of living	15.94	5.56**	15.69	24.05*	17.91
	PwD died	9.06	2.78*	10.78	7.60	14.93
	missing	10.63	11.11	10.78	15.19	4.48

All numbers indicate relative frequencies given in percent except for the values of quality of life of the PwD, which are displayed as the arithmetic mean; numbers in square brackets = range

*SD*  Standard deviation, *iC*   Informal carer, *PwD*  Person with dementia

^a^ *n* = 4 missing values; *indicate significant categories for corresponding types (*p < 0.05, **p < 0.01, ***p < 0.00001)

From the perspective of informal carers, the care situation differed considerably among the four types. The carers of the ‘spouse-centred earlier’ type experienced the care situation as being quite stable. None of the carers in these arrangements stated that caring at home no longer worked, and 45.83% stated that the care situation was well organised and that more help was not needed. In this type, the quality of life of the person with dementia was the highest of all types (32.49). The carers of the ‘spouse-centred later’ and ‘child-centred earlier’ types judged the care situation as less stable. Approximately 10% of them stated that caring at home no longer worked, and more than 60% needed more help in the case of progression of the dementia. The carers of the ‘child-centred later’ type rated their care situation as the least stable. Nearly one-fifth of them (19.40%) stated that caring at home no longer worked. In this type, the quality of life of the persons with dementia was the lowest (26.02).

Furthermore, the living situation of the persons with dementia developed differently in the four types. After one year, 80.56% of the persons with dementia of ‘spouse-centred earlier’ type were still living at home, and only 5.56% had moved to an institutional form of living. The persons with dementia of the types ‘spouse-centred later’ and ‘child-centred later’ moved three times as often (15.69 and 17.91%, respectively) to an institutional form of living. Although the informal carers of the ‘child-centred earlier’ type judged their care as a medium level of stability, the proportion of persons with dementia who moved to an institutional form of living was the highest in this type (24.05%). Only every second person with dementia of this type (53.17%) was still living at home after one year.

## Discussion

Based on the SoCA-Dem theory [15], we identified the underlying structures of differences and commonalities of home-based care arrangements for persons living with dementia. Thus, this study is the first attempt to operationalise the concepts of this theory into measurable variables. Building on these underlying structures, we distinguished four types of home-based care arrangements and compared them with regard to their stability. The four identified types explained 24.08% of the variance between the care arrangements in our sample. This means that there is still a high percentage of variance within the types (75.92%); therefore, the identified types must not be mistaken as being homogenous (see Table 6 and Additional file 1: Appendix E).

‘Dementia and care trajectory’ and ‘structure of the dyadic relationship’ are the two axes that best distinguish care arrangements from each other. The first axis highlights the importance of temporality and change in caring, which is often not adequately recognised in research [34]. Both spouse-centred types and, to a smaller degree, both child-centred types differ significantly from each other with regard to the variables of the SoCA-Dem concept ‘change’. This axis also shows that informal carers in later stages report personal constraints and a loss of relationship with the person with dementia more frequently than informal carers at the earlier stages. However, they also report personal growth more frequently. These relationships were also confirmed in a study by Zank and Schacke [35]. Depending on the position on the ‘dementia and care trajectory’, persons with dementia and informal carers differ in their needs [10, 36, 37], which must be recognised to be able to offer tailored support. In sum, the difference between the two spouse-centred types and between the two child-centred types is grounded mainly in their position on the axis ‘dementia and care trajectory’. Therefore, the question arises whether both ‘earlier’ types will make a transition to become ‘later’ types over the course of time. It is likely that the care dependency as well as the cognitive and functional impairment of the persons with dementia will increase, and the role of the informal carers will change accordingly. Therefore, the care arrangements of both ‘earlier’ types will probably share an increasing number of characteristics with the care arrangements of the ‘later’ types and therefore could transition to the other types. However, not all care arrangements of the earlier types continue long enough to make this transition. For example, 24.05% of persons with dementia of the type ‘child-centred earlier’ moved to an institutional form of living and 7.6% died over the course of one year. The possibility that carers make a transition to other types has also been discussed in other research papers [38, 39]. Since these studies and our study constructed types on the basis of cross-sectional data, the question of transitions between different types cannot be conclusively answered. Therefore, these studies and our results stress the importance of longitudinal research to be able to comprehensively describe and understand care arrangements and their development over time.

The second axis, ‘structure of the dyadic relationship’, separates care arrangements in which the carer is the spouse of the person with dementia from care arrangements in which the carer is an adult child or another family member. Other sociodemographic variables accompany this separation: adult child carers and other family members more often do not live together with the person with dementia, are more often younger than 65 years, and work. The person with dementia for whom they care is often older than 80 years and female. The distinction of care arrangements on the basis of the kinship relation between the carer and the person with dementia is common in research [40, 41]. In addition, our findings show that many other sociodemographic differences accompany this distinction. Expectedly, spouses and adult child carers seem to experience and organise care arrangements differently, which becomes apparent with regard to their stability.

Based on the first 15 axes of MCA, we distinguished four different types of home-based care arrangements. Because the first two axes explained most of the variance, we derived the names of the types from these two axes. Through our naming, we put the main informal carer in the centre of the care arrangements. Although one informal carer often bears the main responsibility of a care arrangement for a person with dementia, our naming could conceal the fact that more persons from the social network, professional carers, and the person with dementia herself/himself often play important roles in these care arrangements [42]. We have already critically discussed this narrow focus on the perspective of one informal carer during the development of our middle-range theory of stability [15]. Nevertheless, our naming of the types affirms this narrow focus instead of expanding it to include, e.g., family dynamics [43] or the perspective of the person with dementia [44] or professional carers [45]. In future research, additional perspectives should be recognised to investigate their impact on the construction of types of care arrangements.

According to our definition of stability, home-based care arrangements are stable if the person with dementia can stay at home and his or her needs and the needs of the informal carer are addressed adequately [11]. The four types of care arrangements differ with regard to the chosen indicator variables for stability. Although the care dependency of the person with dementia and role conflicts of the informal carer in the type ‘child-centred earlier’ are rather low and the care arrangements exist rather briefly, 24.05% of persons with dementia in these care arrangements moved to an institutional form of living within one year. The high rate of institutionalisation in this type is not necessarily an indicator of instability [11]. If the transition to a nursing home is well planned, it might be in the interest of the person with dementia as well as the informal carer [46]. One reason for the high rate of institutionalisation might be that 62% of the persons with dementia in this type live alone, which has been identified as a risk factor for institutionalisation [47] and crises [48]. Persons with dementia often need a high amount of supervision [49], which informal carers who do not share the same household might not be willing or able to provide. In light of the growing spatial distance between informal carers and persons in need of care [50], high integration of women into the workforce and decreasing birth rates in modern societies, the willingness and ability to care might further decrease [51]. A higher utilization of formal support in care arrangements for persons with dementia living alone might be a substitute for the temporary unavailability of the informal carers in these care arrangements [52]. One form of formal support that makes large periods of supervision possible is 24-h live-in carers. Recently, the German Federal Labour Court ruled that live-in carers have a right to the minimum wage even for times of standby duty, which means that they are unaffordable for many families [53]. These societal changes emphasise that the sociocultural context of caring must be taken into account to understand home-based care arrangements for persons with dementia and to support their stability in the best possible way [54]. The SoCA-Dem theory, with its concepts ‘health care system’ and ‘society and culture’ (see Fig. 1), provides a suitable frame for conducting research with this aspiration [15].

In the ‘child-centred later’ type, the rate of institutionalisation over one year was lower (17.91%), although the care dependency and cognitive/functional impairment of the persons with dementia was much higher and the role conflicts of the informal carers were much larger than those of the ‘child-centred earlier’ type. Furthermore, many informal carers of the ‘child-centred later’ type experienced a lack of institutional support, which suggests that supporting a person with dementia living at home is achieved at high costs for the carers of this type, which is a sign of instability. In most cases, the carer was a daughter who cared for her mother and shouldered a large share of care. This finding highlights sex and gender inequalities in informal caring and the need to share care work more equally to relieve caring daughters [55].

Compared to the two child-centred types, both spouse-centred types seem to be more stable. The rate of institutionalisation over one year in the type ‘spouse-centred earlier’ type was very low (5.56%). In the ‘spouse-centred later’ type, the rate of institutionalisation was the second lowest (15.69%). Compared to the ‘child-centred later’ type, the informal carers of the ‘spouse-centred later’ type experienced fewer role conflicts, although the care dependency and functional/cognitive impairment of the persons with dementia was the highest of all types, and almost all care arrangements of this type had been in place for longer than two years. Almost all carers of both spouse-centred types lived together with the person with dementia. Spouses often have high motivation to care for their partner [56]. Living in the same household and not being employed enables continuous company for persons with dementia. However, the often high emotional intimacy and reciprocity of a long-lasting relationship that can be lost during the progression of dementia may also affect spouses to a greater extent than other carers [57]. Having an informal carer who is present for most of the day and highly motivated to care seems to be the reason for the higher stability of spouse-centred care arrangements. Simultaneously, spouse carers tend to integrate other informal and formal carers less often into the care arrangements than child carers do. If a spouse cares for her or his relative mostly alone but is not able to care any longer (e.g., because of his or her own health problems), the stability of these care arrangements is endangered [58]. Early access to formal and informal support might be crucial for spouse-centred care arrangements to keep them stable.

In sum, our results highlight the heterogeneity of home-based care arrangements and underline the importance of recognising and understanding the different and changing needs of persons with dementia and their informal carers during the dementia and care trajectory. In general, spouse-centred care arrangements seem to be more stable than child-centred care arrangements.

Our study is not the first to look for subgroups or types of care arrangements for persons living with dementia. Wiegelmann and colleagues [59] constructed six different classes of care arrangements for persons living with dementia. Similar to our results, the structure of the dyadic relationship was an important distinguishing criterion in their study. In contrast to our results, Wiegelmann et al. identified one class in which the majority of the carers was neither a spouse nor an adult child of the person with dementia. In our results, these care arrangements shared many characteristics with adult–child carers and therefore did not form a separate type in our model. Furthermore, Wiegelmann et al. identified the age and sex of the carer as distinguishing criteria of care arrangements with a spouse as the carer. Although the sex of the carer could influence the experience of carers [17, 60], in our model, it did not correspond strongly with other included variables (see Additional file 1: Appendix C) and therefore did not lead to a separate type.

Janssen and colleagues constructed five different profiles of carers of persons with dementia [61]. They argue that it might be possible to categorise carers of persons with dementia along two axes. The first axis includes the age of the carer, the kinship relation between the carer and the person with dementia and the severity of dementia. The combination of aspects of the dyadic relationship with the progression of dementia into one axis of differences is not supported by our results as both aspects are related to different axes in our model. Their second axis includes the tendency towards stress and difficulties in adapting to stress. We did not include variables to measure the ability of the carer to adapt to stress. Therefore, we were unable to differentiate care arrangements along a corresponding axis. In the SoCA-Dem theory [15], the ability to adapt is part of the concept of ‘resources’ and influences the stability of home-based care arrangements. Therefore, it would be worthwhile to include appropriate variables in future research. Nevertheless, the adaptability of the carer is only one of many elements that influences the stability of home-based care arrangements [62].

Overall, the studies of Wiegelmann et al. and Janssen et al. as well as our study highlight that care arrangements for persons living with dementia are heterogeneous. The structure of the dyadic relationship is one important distinguishing criterion of home-based care arrangements identified in all studies.

### Strengths and limitations

The SoCA-Dem theory is a suitable framework to guide the selection of relevant variables to construct different types of care arrangements. Our use of the SoCA-Dem theory as a theoretical framework is a major strength of the present study because it justifies the selection of variables. However, since our study is a secondary analysis, we were unable to include variables for all concepts of the entire SoCA-Dem theory. This could also be one reason for the high percentage of variance within the types. In future research, the concepts of the SoCA-Dem theory need to be operationalized. For instance, we were not able to include the ability of the informal carer to adapt and cope with stress (included in the concept ‘resources’ of the SOA-Dem theory), the quality of the relationship between the person with dementia and the informal carer (included in the concept ‘dyadic relationship’ of the SoCA-Dem theory), or the motivation of the informal carer to assume the carer role (included in the concept ‘carer role’ of the SoCA-Dem theory).

Our analytical approach – multiple correspondence analysis in combination with hierarchical cluster analysis – has the strength of a visual representation of the results, which enables a detailed interpretation of the structure of the data. However, the MCA requires categorical data with preferably only a few categories. Therefore, we transformed some continuous variables and many variables with more than two answer categories into variables with predominantly two answer categories. This transformation implies a reduction of information that may have shaped our results. In particular, the axis ‘dementia and care trajectory’ is formed by variables that are based on variables that initially have many answer categories or even a continuous level of measurement. Our four types of care arrangements are positioned along this axis. The clear border between the four types in relation to the ‘dementia and care trajectory’ might be influenced by our transformation of the data and might partly conceal the more continuous character of the dementia and care trajectory. However, intensification into a few categories and the accompanying reduction in complexity could be seen as aligned with the principle of parsimony, which helps to understand the underlying structures [63].

A final limitation is related to the representativeness of our sample and the generalizability of our results. The sample of the DemNet-D study was a convenience sample. To be included in the DemNet-D study, a care arrangement already had to be supported by a dementia care network and, hence, by the formal care system. Care arrangements at the very beginning of the dementia and care trajectory or without support from formal care providers might have been underrepresented in the DemNet-D study. Nevertheless, Wolf-Ostermann and colleagues [18] argued that the sample of the DemNet-D study is a valid representation of home-based care arrangements in Germany. Our study is a secondary analysis of the DemNet-D study, and our sample does not differ greatly from the entire DemNet-D sample. In comparison to the representative sample of the MUG-III study [64], in our sample, more men living with dementia and spousal carers were included. For a detailed comparison between our sample, the DemNet-D sample and the MUG-III study, see Additional file 1: Appendix F. The generalizability of our results is limited. It is possible that care arrangements at the beginning of showing dementia symptom or diagnose and at the beginning of the care trajectory and/or without contact to the formal health care system could form other types of care arrangements. Furthermore, the size of the types need to be interpreted with caution as spousal carers might be overrepresented in our sample. Finally, the study was conducted in Germany with its specific health care system, demography and social norms. It is possible that in other health care systems or countries the size of the types differs from our results or other types could be constructed.

### Implications for research and practice

In research, persons with dementia and their informal carers are often constructed as a homogenous group with a unifying problem: dementia [5]. This conceptualisation neglects to recognise the heterogeneity of home-based care arrangements and could be one reason for the rather moderate evidence of effective interventions [8]. In response to this gap in the research, the effects of interventions are increasingly studied in subgroups divided, for instance, by gender, family relationship, ethnicity or dementia severity [6]. Our types have the advantage of simultaneously capturing several dimensions of differences and commonalities and thus point to possible interactions between important variables that are associated with the stability of care arrangements. Therefore, our results could be used to study the effects of interventions for different types of care arrangements.

However, interventions are predominantly not tailored to the needs of specific subgroups [65]. In the future, the effects of interventions should not only be studied for different types of care arrangements but interventions should also be specifically tailored to the different needs of these types of persons. Although our four types cannot capture the complexity of the individual situation of a specific care arrangement [42], they can help to guide the development of tailored interventions by better explaining and distinguishing care arrangements. However, this will not supersede the need for individual assessments of the needs of the involved persons [66] to tailor interventions and to deliver person-centred care [67].

## Conclusion

Home-based care arrangements for persons living with dementia are heterogeneous but also share commonalities. Guided by the SoCA-Dem theory, we constructed four types of care arrangements for persons with dementia living at home who are cared for by an informal carer. Our results contribute to a detailed description of care arrangements and a better understanding of their underlying structures. We constructed two spouse-centred and two child-centred types that differ with regard to the ‘structure of the dyadic relationship’ and their position on the ‘dementia and care trajectory’. Furthermore, we provided the first indication that these care arrangements also differ in their stability. Our results highlight the need for research to better acknowledge and understand the heterogeneity of care arrangements while designing and testing interventions. Despite our results, there is still a need to grasp the complexity of home-based care arrangements, their development over the course of time and their embeddedness in the specific social context.

## Supplementary Information

**Additional file 1:** **Appendix A.** Variables of the present study and the underlying variables of the DemNet-D-Study. **Appendix B.** The eigenvalues an their percentage of explained variance. **Appendix C.** Plot of squared cosine values. **Appendix D.** Dendrogram. **Appendix E.** Silhouette plot. **Appendix F.** Comparison between the sample of the present study, the DemNet-D study and the representative MUG III study.

## Abbreviations

DCN: Dementia Care Networks
DZNE: Deutsches Zentrum für Neurodegenerative Erkrankungen
HCA: Hierarchical Cluster Analysis
iC: Informal Carer
MCA: Multiple Correspondence Analysis
PwD: Person with Dementia
SoCA: Stability of Home-Based Care Arrangements

## Declarations

### Availability of data and materials

The publication of the data in a public data repository is not possible, because of restriction of the informed consent. The data can be accessed after request at the DZNE site Witten, Germany. Please contact the data management of the DZNE site Witten (data-management-witten@dzne.de).
