# Supplementary material for: Differences and commonalities of home-based care arrangements for persons living with dementia in Germany – a theory-driven development of types using multiple correspondence analysis and hierarchical cluster analysis
Source: BMC Geriatr. 2022 Sep 1;22:723. doi: 10.1186/s12877-022-03310-1 (PMC9438141; doi:10.1186/s12877-022-03310-1)
Supplement: Supplementary file 1 — Additional file 1: Appendix A. Variables of the present study and the underlying variables of the DemNet-D-Study. Appendix B. The eigenvalues an their percentage of explained variance. Appendix C. Plot of squared cosine values. Appendix D. Dendrogram. Appendix E. Silhouette plot. Appendix F. Comparison between the sample of the present study, the DemNet-D study and the representative MUG III study. [file 12877_2022_3310_MOESM1_ESM.docx]

**Appendix**

[Appendix A: Variables of the present study and the underlying variables of the DemNet-D-Study 2](#_Toc77261735)

[Appendix B: The eigenvalues an their percentage of explained variance 5](#_Toc77261736)

[Appendix C: Plot of squared cosine values 6](#_Toc77261737)

[Appendix D: Dendrogram 7](#_Toc77261738)

[Appendix E: Silhouette plot 8](#_Toc77261739)

[Appendix F: Comparison between the sample of the present study, the DemNet-D study and the representative MUG III study. 9](#_Toc77261740)

# Appendix A: Variables of the present study and the underlying variables of the DemNet-D study

**Active variables**

| **Concept** | **Variable** | **Categories** | **Categories of the underlying DemNet-D variables** |
| --- | --- | --- | --- |
| **Dyadic Relationship** | kinship relation between the iC and the PwD | spouse/partner | iC is the spouse/partner of the PwD |
|  |  | parent–child | iC is the daughter/son of the PwD |
|  |  | other relative | iC is a grandchild OR child-in-law OR other family member OR neighbour OR friend of the PwD |
|  | living situation of the PwD | living alone (or alone with a 24 h help) | PwD lives alone OR the PwD lives alone with 24-h help |
|  |  | cohabiting with iC | (PwD lives with spouse OR children/children in law OR in own household but same house as a relative OR together with another relative) AND the iC lives together with the PwD in one household |
|  |  | cohabiting with another relative | (PwD lives with spouse OR children/children in law OR in own household but same house as a relative OR together with another relative) AND the iC does not live together with the PwD in one household |
|  | loss of relationship between the PwD and the iC | often/always | three of five items of the BICS-D [1] subscale loss of relationship = 0-6 |
|  |  | never/sometimes | three of five items of the BICS-D subscale loss of relationship = 7-12 |
|  | age of the iC | up to 65 years old | age of iC in years ≤ 65 years |
|  |  | older than 65 years old | age of iC in years > 65 years |
|  | age of the PwD | up to 80 years old | age of iC in years ≤ 80 years |
|  |  | older than 80 years old | age of iC in years > 80 years |
| **Change** | care level of the PwD according to the German long-term care insurance | no care level | no care level OR applied for a care level, not decided OR applied for a care level, not approved |
|  |  | first care level | care level 1 |
|  |  | second or third care level | care level 2 OR care level 3 |
|  | functional/cognitive ability of the PwD (FAST) | until moderate Alzheimer’s | up to stage 5 of the FAST [2] |
|  |  | moderately severe Alzheimer’s | stage 6a to stage 6e of the FAST |
|  |  | severe Alzheimer’s | stage 7a to stage 7f of the FAST |
|  | period since the PwD needs help because of the memory loss | since up to 2 years | PwD needs help because of memory loss from up to 3 months OR from more than 3 months up to 6 months OR from more than 6 months up to 1 year OR from more than 1 year up to 2 years |
|  |  | since more than 2 yeas | PwD needs help because of memory loss from more than 2 years up to 3 years OR from more than 3 years up to 4 years OR from more than 4 years up to 5 years OR from more than 5 years |
|  | period since the iC cares for the PwD | since up to 2 years | involvement of iC in care for PwD in years and months ≤ 2 years |
|  |  | since more than 2 years | involvement of iC in care for PwD in years and months > 2 years |
| **Carer Role** | role conflict between caring for the PwD and profession | always/often | (iC is working fulltime OR part-time OR on an hourly base) AND BICS-D subscale *work role conflicts* = 0-8 |
|  |  | never/sometimes | (iC is working fulltime OR part-time OR on an hourly base) AND BICS-D subscale *work role conflicts* = 9-16 |
|  |  | not working | iC is not working |
|  | the iC experiences personal constraints due to caring | always/often | five of nine items of the BICS-D subscale *personal constraints/health* = 0-10 |
|  |  | never/sometimes | five of nine items of the BICS-D subscale *personal constraints/health* = 11-20 |
|  | the iC experiences personal growth due to caring | always/often | BICS-D subscale *personal growth* = 0-10 |
|  |  | never/sometimes | BICS-D subscale *personal growth* = 11-20 |
|  | the iC experiences no recognition from others | always/often | two of four items of the BICS-D subscale *lack of social recognition* = 0-4 |
|  |  | never/sometimes | two of four items of the BICS-D subscale *lack of social recognition* = 5-8 |
|  | the iC could imagine the PwD moving to institutional care | yes | iC could imagine PwD moving to an assisted living facility OR a nursing home OR shared living for PwD |
|  |  | no | iC could not imagine PwD moving to an assisted living facility OR a nursing home OR shared living for PwD |
| **Resources** | number of professional services used | none | PwD does not use an ambulant nursing service OR day care OR support group OR visiting service OR respite care OR meals-on-wheels |
|  |  | as least one | PwD does use an ambulant nursing service OR day care OR support group OR visiting service OR respite care OR meals-on-wheels |
|  | number of groups of informal supporters | one | one of the following groups is involved in the care arrangement: spouse of PwD, children of PwD, other relatives of PwD, neighbours of PwD, friends |
|  |  | two or more | more than one of the following groups is involved in the care arrangement: spouse of PwD, children of PwD, other relatives of PwD, neighbours of PwD, friends |
|  | the PwD or the iC has sufficient financial resources | yes | iC or PwD has sufficient financial resources |
|  |  | no | iC or PwD has not sufficient financial resources OR the iC OR the PwD has to pay attention to their financial resources |
| **Society & Culture** | gender of the iC | female | gender of the iC is female |
|  |  | male | gender of the iC is male |
|  | gender of the PwD | female | gender of the PwD is female |
|  |  | male | gender of the PwD is male |
|  | migration background of the iC | yes | place of birth of the mother of the iC is not Germany OR place of birth of the father of the iC is not Germany |
|  |  | no | (place of birth of the mother of the iC is Germany OR unknown) AND (place of birth of the father of the iC is Germany OR unknown) |
|  | the iC experiences too little understanding of others for the PwD | always/often | two of four items of the BICS-D subscale *lack of social recognition* = 0-4 |
|  |  | never/sometimes | two of four items of the BICS-D subscale *lack of social recognition* = 5-8 |
| **Health Care System** | the iC experiences a lack of institutional support | always/often | BICS-D subscale *lack of institutional support* = 0-6 |
|  |  | never/sometimes | BICS-D subscale *lack of institutional support* = 7-12 |
| **Balancing** | - | | no suitable variables could be identified in the DemNet-D dataset |
| **Needs** | - | | no suitable variables could be identified in the DemNet-D dataset |

**Passive variables**

| **Variable** | **Answer Categories** | **Categories of the underlying DemNet-D variables** |
| --- | --- | --- |
| care situation from the perspective of the iC at t0 | caring at home does not work anymore | caring at home does not work anymore, we are thinking about moving PwD to a nursing home OR caring at home does not work anymore, we need additional help to keep on caring at home |
|  | the care situation is well organised, but in case of progression of the dementia, more help is needed | the care situation is well organised, but in a case of a progression of the dementia, more help is needed |
|  | the care situation is well organised; even if the dementia progresses, no more help is needed | the care situation is well organised, even if the dementia progresses no more help is needed |
|  | missing | missing |
| quality of life of the PwD at t0^a^ | | QOL-AD (proxy, iC) [3] |
| living situation of the PwD at t1 | still private home | PwD lives alone OR alone with 24-h help OR with spouse OR children/children in law OR in own household but same house as a relative OR together with another relative |
|  | PwD lives in an institutional form of living | PwD lives at a nursing home OR in an assisted living facility OR in a shared flat for PwD OR PwD moved to a nursing home OR to an assisted living facility OR in a shared flat for PwD |
|  | PwD died | PwD died |
|  | missing | missing |

BICS-D = Berlin Inventory of Caregiver Stress—Dementia; FAST = functional assessment staging; iC = informal carer; PwD = person with dementia; QOL-AD = quality of life Alzheimer’s Disease; “OR” and “AND” are used as Boolean operators to combine answer categories of the DemNet-D study into new categories for our secondary analysis

References

1. Schlomann A, Schacke C, Leipold B, Zank S: Berlin Inventory of Caregiver Stress—Dementia (BICS-D) The Gerontologist 2020:1-12, 10.1093/geront/gnz195.

2. Reisberg B: Functional assessment staging (FAST) Psychopharmacol Bull 1988, 24(4):653-659.

3. Logsdon RG, Gibbons LE, McCurry SM, Teri L: Assessing Quality of Life in Older Adults With Cognitive Impairment Psychosomatic Medicine 2002, 64(3):510-519, 0033-3174/02/6403-0510.

# Appendix B: The eigenvalues and their percentage of explained variance


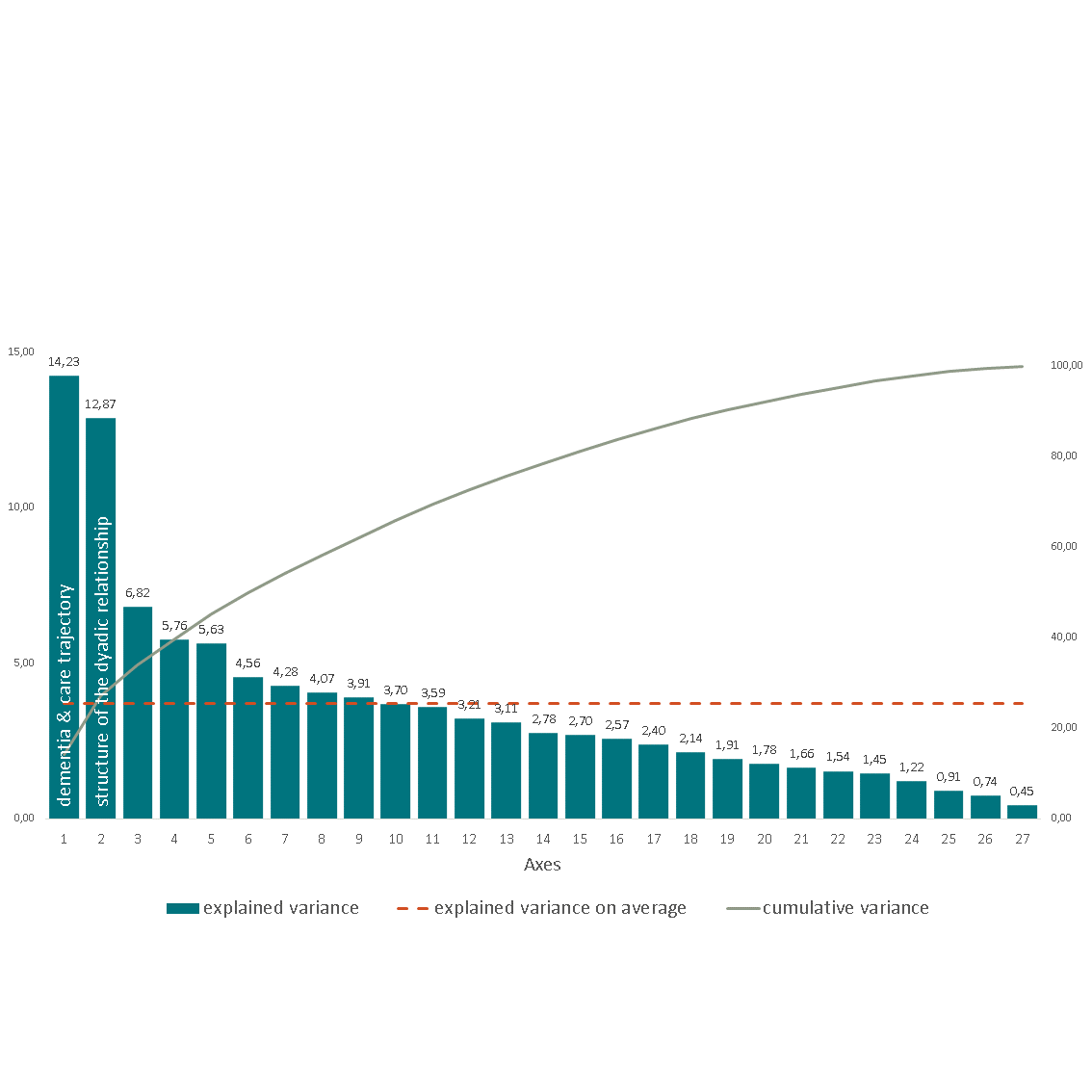


# Appendix C: Plot of squared cosine values

The quality of representation of all categories for all axes can be displayed with the squared cosine plot. The darker and larger the circle of one category is, the better the representation of the category by the corresponding axis.
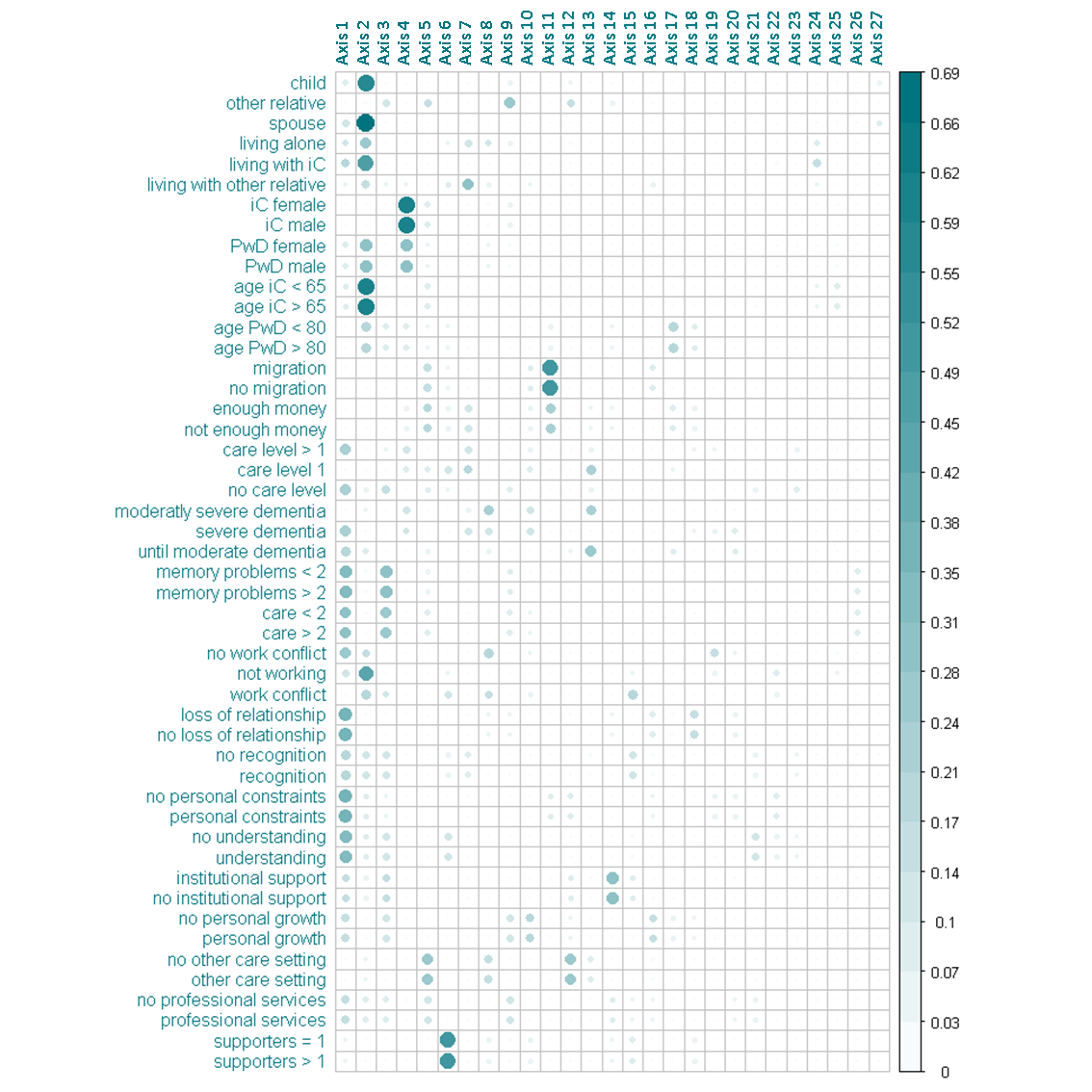


Short names are explained in table 2 of the main document.

# Appendix D: Dendrogram


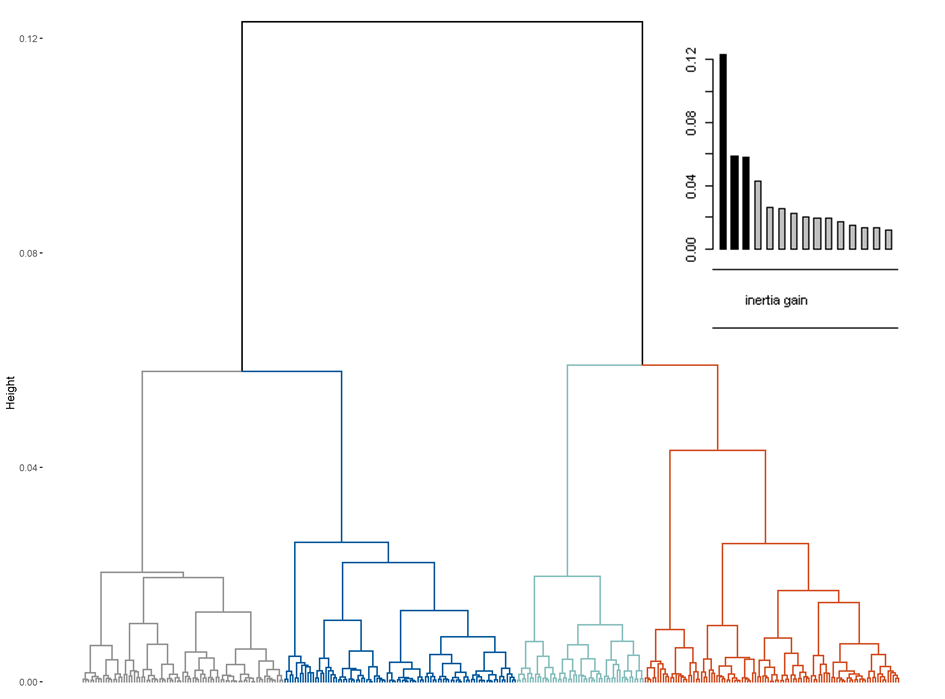


#
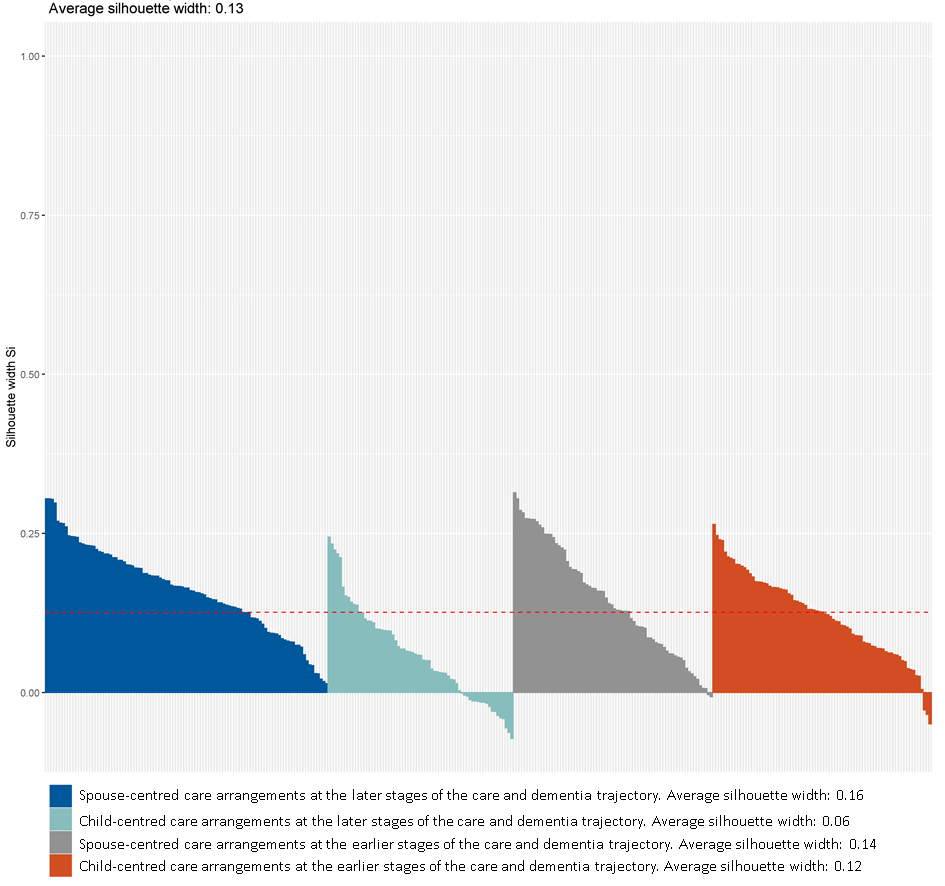
Appendix E: Silhouette plot

# Appendix F: Comparison between the sample of the present study, the DemNet-D study and the representative MUG III study.

| Sample characteristics | Sample of the present study  (n=320) | DemNet-D study t0  (n=560) [1] | MUG III study  (n=151) [2] |
| --- | --- | --- | --- |
| Gender of the person with dementia  Female  Male  Missing | 56.3  43.8  0 | 58  41.4  0.5 | 69.5  30.5  0 |
| Age of the person with dementia | 79.62 (SD ± 8.17), [44-101]* | 79.74 (SD ± 8.42), [44-103] | 81 (SD ± 7.9), [62-102] |
| Living situation of the person with dementia  Cohabiting with spouse/partner in same household  Living alone in own household  Cohabiting with adult children (in-law) in same household  In a relative’s place but in own independent household  Cohabiting with other relative in same household  Living alone in own household with live-in carer  Not a private form of living | 56.9  21.9  11.6  7.8  1.6  0.3  0 | 51.6  20.7  8  7  1.4  1.3  9.8 | NA  NA  NA  NA  NA  NA  NA |
| Living in the same household as the primary informal carer  Yes  No  Missing | 68.1  31.6  0.3 | 60.2  38.6  1.3 | 73.6  NA  NA |
| Diagnoses of dementia by a physician  Yes  No  Missing | 91.3  6.6  2.2 | 89.8  7  3.2 | 31.3  NA  NA |
| Dementia aetiology  Alzheimer’s dementia  Vascular dementia  Fronto-temporal dementia  Dementia with Lewi bodies  Parkinson’s dementia  Unspecific  Missing | 38.8  18.4  0.9  0.3  1.3  27.8  12.5 | 37.7  16.4  1.3  0.4  1.1  27.7  15 | 28.3  10.9  NA  NA  NA  60.9  NA |

| Sample characteristics | Sample of the present study  (n=302) | DemNet-D study t0  (n=560) | MUG III study  (n=151) |
| --- | --- | --- | --- |
| Care level  None  Care level 1  Care level 2  Care level 3  Missing | 25.4  40.3  28.7  5.6  0 | 23.7  35.9  28.9  6.1  5.4 | 24.8  32.2  29.5  13.4  0 |
| Kinship relation between the person with dementia  and the informal carer  Spouse  Child  Child-in-law  Other family member  Friend  Professional carer  Missing | 54.4  37.2  5  2.5  0.9  0  0 | 50  36.8  3.8  2.9  1.1  5.4  0.2 | 38.5  37.7  13.2  8  0.7  0  2 |
| Gender of the informal carer  Female  Male  Missing | 75.3  24.7  0 | 74.5  24.8  0.7 | 73  NA  NA |
| Age of the informal carer | 64.53 (SD ± 12.5), [24-93] | 63.88 (SD ± 12.91), [24-93] | 61.3 (SD ± 14.3),[NA] |
| Migration background of the informal carer  Yes  No  Missing | 8.1  91.9  0 | 10.2  88.2  1.6 | NA  NA  NA |
| Employed  Yes  No  Missing | 33.1  66.9  0 | 35.7  62.5  1.8 | 26.9  NA  NA |

References

1. DemNet-D Consortium: Multizentrische, interdisziplinäre Evaluationsstudie von Demenznetzwerken in Deutschland (DemNet-D). Sachbericht des Forschungsverbundes [Multicenter, interdisciplinary evaluation study of dementia networks in Germany (DemNet-D). Report of the research consortium]. Greifswald; 2016.
2. Schneekloth U, Wahl HW: Möglichkeiten und Grenzen selbständiger Lebensführung in privaten Haushalten (MUG III). Repräsentativbefunde und Vertiefungsstudien zu häuslichen Pflegearrangements, Demenz und professionellen Versorgungsangeboten. Integrierter Abschlussbericht [Possibilities and limits of independent living in private households (MUG III). Representative findings and in-depth studies on home care arrangements, dementia, and professional care services. Integrated final report]. München; 2005.
